# Determinants of Perceived Comfort: Multi-Dimensional Thinking in Smart Bedding Design

**DOI:** 10.3390/s24134058

**Published:** 2024-06-21

**Authors:** Xiangtian Bai, Yonghong Liu, Zhe Dai, Yongkang Chen, Pingping Fang, Jun Ma

**Affiliations:** 1 School of Design, Hunan University, Changsha 410082, China; xtbai@hnu.edu.cn (X.B.); dzqw2016@gmail.com (Z.D.); pingpingping0606@gmail.com (P.F.); 2 Innovation Institute of Industrial Design and Machine Intelligence, Hunan University, Quanzhou 362006, China; 3 College of Design and Innovation, Tongji University, Shanghai 200092, China; 2210919@tongji.edu.cn; 4 Xiangya School of Nursing, Central South University, Changsha 410013, China; majun_cs@csu.edu.cn

**Keywords:** perceived comfort, body pressure, smart bedding, sleep posture, sensor application

## Abstract

Sleep quality is an important issue of public concern. This study, combined with sensor application, aims to explore the determinants of perceived comfort when using smart bedding to provide empirical evidence for improving sleep quality. This study was conducted in a standard sleep laboratory in Quanzhou, China, from March to April of 2023. Perceived comfort was evaluated using the Subjective Lying Comfort Evaluation on a seven-point rating scale, and body pressure distribution was measured using a pressure sensor. Correlation analysis was employed to analyze the relationship between perceived comfort and body pressure, and multiple linear regression was used to identify the factors of perceived comfort. The results showed that body pressure was partially correlated with perceived comfort, and sleep posture significantly influenced perceived comfort. In addition, height, weight, and body mass index are common factors that influence comfort. The findings highlight the importance of optimizing the angular range of boards based on their comfort performance to adjust sleeping posture and equalize pressure distribution. Future research should consider aspects related to the special needs of different populations (such as height and weight), as well as whether users are elderly and whether they have particular diseases. The design optimization of the bed board division and mattress softness, based on traditional smart bedding, can improve comfort and its effectiveness in reducing health risks and enhancing health status.

## 1. Introduction

Sleep quality is an important social issue of public concern [1]. Good sleep quality not only helps people recover from physical exhaustion, but also largely contributes to psychological health, enhances well-being, and improves one’s overall quality of life [2,3]. However, the sleep quality of Chinese citizens has declined in recent years. The Sleep Research Report of China, published in 2023, showed that the average nightly sleep duration of Chinese citizens was 8.25, 7.78, 7.69, and 7.16 h in 2010, 2014, 2016, and 2018, respectively [4]. More than 50% of citizens under age 45 have sleep problems, and more than 40%—even among those aged 25 and younger—have sleep problems [4]. Improving the sleep quality of the public requires collaboration between a multidisciplinary team of researchers in the fields of medicine, psychology, and design.

Sleep occupies 30% of one’s general time, and creating a comfortable and relaxing bedding space is the first essential element of high-quality sleep [5]. In the sleep scene, consumers focus on creating a healthy, quiet, and comfortable bedroom (mattress/bed) at home, and bedding is still the focus of consumption; in addition, young consumers also focus on intelligent bedding to monitor and enhance sleep quality [6]. Thus, the comfort provided by smart bedding has become a hot topic in the field of home product design [7,8].

The main structure of traditional bedding consists of four parts: bed boards, bed frames, mattresses, and bedspreads. Traditionally, bedding comfort has been influenced by bed width, mattress softness, and other physical indicators [9]. Hence, designers should focus on the softness and support of mattresses when considering bedding comfort [10]. Smart bedding is based on a traditional bed structure with the addition of adjustable bed boards and software control systems [11]. Product features of smart bedding include unique sensing technology, the ability to adjust the bed’s posture [12,13], smart medical home carriers, high-quality carriers for real-time sleep monitoring, the ability to prevent and manage health problems, the capacity to accumulate significant quantities of data on sleep [14], and other advantageous functions in mobile health management. In the design process of smart bedding, the bed board can be flexibly adjusted through the intelligent control of the software system to adapt to the unique needs of different groups for special sleeping postures [15,16], to enhance comfort, and to help people obtain quality sleep [17].

Some researchers have assessed physiological parameters (such as mean and peak pressure) when designing other products for sitting and lying down and combined them with subjective comfort ratings to collectively understand the perceived comfort of the product [18,19,20,21,22,23,24]. Dangal and colleagues designed and developed a prototype aircraft seat pan using spring-foam technology, and utilized questionnaires and pressure chart recordings in a comparative evaluation of the test against a standard aircraft seat pan. It was found that the pressure distribution on the prototype seat pan was significantly closer to an ideal pressure distribution, and was also lighter and more comfortable than conventional foam cushioning materials [18]. Li and colleagues found a correlation between the distribution of seat interface pressure and the perceived comfort of vehicle occupants, and that by developing regression models based on pressure distribution data, the comfort rating experienced during driving can be effectively analyzed, and this approach can enhance the accuracy and effectiveness of research and evaluation of car seat comfort [19]. Yao explored the relationship between pressure maps and the comfort/discomfort of users in aircraft seats, while proposing a new six-point method on pressure maps collected at the bottom of the cushion [21]. Huang and colleagues analyzed the pressure distribution and changes in sitting comfort of high-speed train second-class seats with different design parameters, especially the influence of the seat angle on the interface pressure distribution and comfort, and found that the interface pressure distribution on the seat cushion and backrest become more manageable as the seat inclination angle increases within 10 degrees [23]. In addition to factors such as contact surface contour and thickness, some of the above studies have addressed the effect of angle on body pressure distribution and perceived comfort, with better results and greater help in design iterations. However, such studies on smart bedding designs are limited and have not been conducted based on smart bedding product characteristics. Existing studies on bedding comfort usually understand product comfort in terms of pressure perception. For example, Ren et al. quantitatively analyzed how the layered structure of mattress bedding affected pressure relief performance and subjective lying comfort by investigating mattress support performance, interface pressure distribution, and subjective lying comfort; the results showed that pressure relief performance is an important factor influencing the comfort score [25]. Park and colleagues investigated the relationship between subjective sleep comfort and bed adjustment, and found that the hip/thigh area was under the most pressure, and, based on objective criteria, this area was the most uncomfortable [26]. The interactive bedding system designed by Jun Ito incorporates an innovative approach to body pressure dispersion while considering various body shapes and sleeping positions. The proposed method ensures ideal pressure distribution across the bed’s surface, aiming to enhance sleep comfort [27]. In addition, bedding comfort varies at the level of individual subjective characteristics, such as demographic traits (e.g., sex, age, and body mass index [BMI]) [28,29] and psychosocial status (e.g., perceptual states, mindfulness, and experience) [30]. It is noteworthy that the boundary between seats and bedding is becoming increasingly blurred. Along with the profound transformation of fully automated driving in the automotive industry, its interior design is also undergoing major changes. Car seats can also be adjusted for multiple angles of the back, head, legs, and even overall zero-gravity seating, and users are increasingly choosing to sleep while traveling [31]. As an example, the 2024 Li L9 launched by Li Auto Inc. features a second-row right seat that can recline backward to a maximum angle of 140 degrees, and the leg rests and footrests will slowly open up, resulting in a corresponding reduction in pressure on the torso and hips [32]. Many types of seat comfort research methods can be applied to bedding comfort research. Therefore, the combination of objective measurement data and evaluations of subjective comfort provides a new perspective for more accurately testing the comfort of smart bedding and optimizing product design.

As such, we experimentally tested the body pressure distribution of groups with different individual features when using smart bedding, collected subjective comfort scores simultaneously, and analyzed subjective and objective indicators to provide more comprehensive, accurate empirical evidence for the design of smart beddings and the improvement of the public’s sleep quality.

## 2. Materials and Methods

We wrote this article according to the Strengthening the Reporting of Observational Studies in Epidemiology 2007 (STROBE 2007) [33].

### 2.1. Study Design and Settings

**Figure 1:**
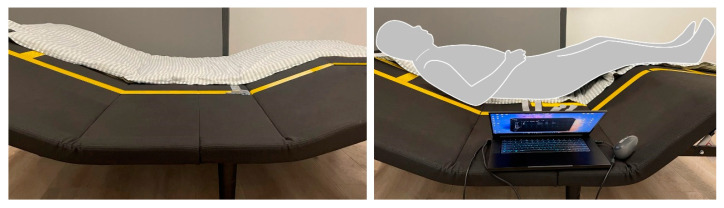
Smart bed with pressure sensor mat.

We conducted this cross-sectional study to assess body pressure and perceived comfort during smart bedding use in a standard sleep laboratory at the Quanzhou Innovation Research Institute of Hunan University from March to April of 2023. The laboratory provided a stable experimental environment, ensuring the consistency of factors such as temperature, sound, humidity, and light during data collection. The smart bed used is a prototype that effectively represents the main features of smart bedding, including the bed board adjustment function, while omitting massage, sleep aids, and respiratory monitoring to prevent added complexity (Figure 1). This basic smart-bed prototype consists of a stainless-steel bed frame and four adjustable bed boards linked by pivots. The bed boards are 180 cm long on one side and vary in length (65, 50, 32, and 40 cm) on the other side, all with a thickness of 20 mm. Driven by electric actuators, these bed boards can pivot, allowing for the smart bed to be adjusted to different conditions. Table 1 outlines the typical conditions for the smart bed’s angle limit setting. The pressure distribution test was carried out using the Tekscan BRE5315 textile pressure measure systems (Tekscan, Inc., South Boston, MA, USA). The experimental pressure-sensing mat consisted of 8064 sensing elements and covered an area of 42.67 × 195.07 cm (Figure 1). The measuring range of its sensors is from 0 to 33.92 kPa.

### 2.2. Participants

We used convenience sampling to recruit 408 participants from an industrial park in Quanzhou, Fujian, China. The inclusion criteria were as follows: (1) participants had used at least two types of smart health products, such as massage chairs, neck pillows, and treadmills; and (2) each participant must have used a smart health product within the past two months [34]. The exclusion criteria were as follows: (1) participation in similar studies in the past; and (2) severe physical or mental illness and the inability to cooperate with the investigation. We sequentially numbered the participants included in the experiment for identity labeling, and then used IBM SPSS statistical software (version 26.0; Armonk, NY, USA) to generate sequences of random numbers. Participant numbers were allotted in correspondence with the random numbers, and we arranged the random numbers in descending order to group participants into groups of 40 participants each. Thus, the recruited participants were randomly divided into ten posture-based groups. Each participant corresponded to testing only one typical bed condition.

### 2.3. Variables and Measurement

**Figure 2:**
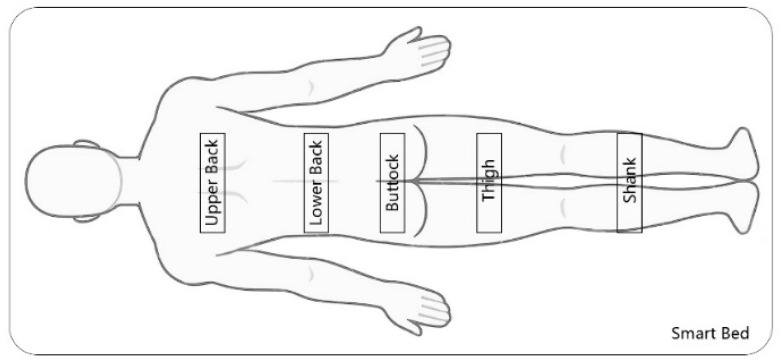
Contact area division between the body and the smart bed.

The rating scale is one of the most fundamental and frequently used tools for evaluating subjective comfort [25]. We assessed perceived comfort using the Subjective Lying Comfort Evaluation on a seven-point Rating Scale [35], where −3 indicates the least comfortable and +3 denotes the most comfortable. The seven-point Likert scale strikes a suitable balance, allowing for sufficient discrimination in responses without an overwhelming number of options [36,37]. To study the variation of comfort in different partitions of the body, based on the smart bed board arrangement, we broadly divided the body into four regions: the upper back, the lower back, the buttocks and thigh, and the shank area (Figure 2) [38]. Consequently, the assessment items included: *“Please assess the present overall comfort level of your body,” “Please assess the present comfort level of the upper back area,” “Please assess the present comfort level of the lower back area,” “Please assess the present comfort level of the buttocks and thigh area,” and “Please assess the present comfort level of the shank area”.*

We assessed body pressure using a pressure sensor positioned on the bed to capture the body pressure distributions (Figure 1). We recorded real-time pressure data for each sensor element, and saved the data matrix in Excel. For each test, we employed the pressure measurement software to compute the overall pressure, contact area, mean pressure, and peak pressure for each participant in the corresponding smart bed condition. We determined the overall pressure, contact area, and peak pressure via direct measurements. Let *k* be generic occupants, and let $P_{ij}^{k}$ represent the pressure over cell *ij* of the sensor matrix for participant *k*. Correspondingly, $S^{k}$ is the contact area where the sensor matrix pressure is not zero. We calculated the mean pressure $\bar{P^{k}}$ as follows:

$$\bar{P^{k}}=\sum_{i=1,j=1}^{I,\;J}P_{ij}^{k}/S^{k},$$

where *I*, *J* is the total number of sensor cells, and where 1 ≤ *i* ≤ 50, 1 ≤ *j* ≤ 250.

### 2.4. Procedure

Before the experiment, we obtained written informed consent from all participants. Eligible participants were thoroughly briefed on the procedures and were asked to fill out a questionnaire about general information, including their sex, age, height, and weight. Accordingly, we calculated the participants’ BMIs. The research assistant randomly led each participant into the laboratory and blinded them to a corresponding bed condition. Participants were asked to lie flat on their back with specific instructions, as follows: The buttocks were positioned in the middle of the bed board, while the head, back, and legs were naturally extended in the supine position. The smart bed was in a fully flat condition at the beginning of each experiment. After the participants had been lying flat for more than 20 min, the smart bed was adjusted to the typical conditions corresponding to that participants’ grouping. Participants were informed that they could freely adjust their sleeping posture while lying in the same position. After maintaining the typical conditions for 30 min, the smart bed was returned to a fully flat condition. During this period, participants’ body pressure indicators were recorded by the pressure measurement system, and the research assistant asked them about their perceived comfort level and recorded the responses.

### 2.5. Statistical Data Analysis

We imported data into the IBM SPSS statistical software (version 26.0) for analysis. We only used complete data on the included variables. We calculated summary descriptive statistics using appropriate measures of central tendency (mean) and dispersion (standard deviation [SD]) for continuous data, and number and percentage for categorical data. We used an analysis of variance (ANOVA) to compare the differences in perceived comfort between the ten groups, and further used the Bonferroni method for two-by-two comparisons, and employed correlation analysis to analyze the relationship between somatic pressure and perceived comfort. We used multiple linear regression to examine the effects of individual demographic factors on body pressure and perceived comfort. We considered all statistical tests to be “significant” for a *p*-value ≤ 0.05.

## 3. Results

### 3.1. Descriptive Statistics

We initially recruited 420 participants, of whom 408 were eligible to participate. After excluding individuals with incomplete data (*n* = 8), the final sample size was 400. Table 2 presents descriptive statistics for the 400 participants, of whom 64% were male and 36% were female. They ranged in age from 23 to 35 years old, with a mean (SD) age of 28.28 (3.41) years. Their weight ranged from 42.2 kg to 85.2 kg, with a mean (SD) weight of 65.10 (16.50) kg. Their height ranged from 152.0 cm to 188.5 cm, with a mean (SD) height of 169.36 (9.13) cm. Their BMI value ranged from 16.82 kg/m^2^ to 30.77 kg/m^2^, with a mean (SD) BMI value of 22.39 (3.95) kg/m^2^. Differences in demographic traits between the groups were not statistically significant.

### 3.2. Main Results

#### 3.2.1. Relationship between Body Pressure and Perceived Comfort

**Figure 3:**
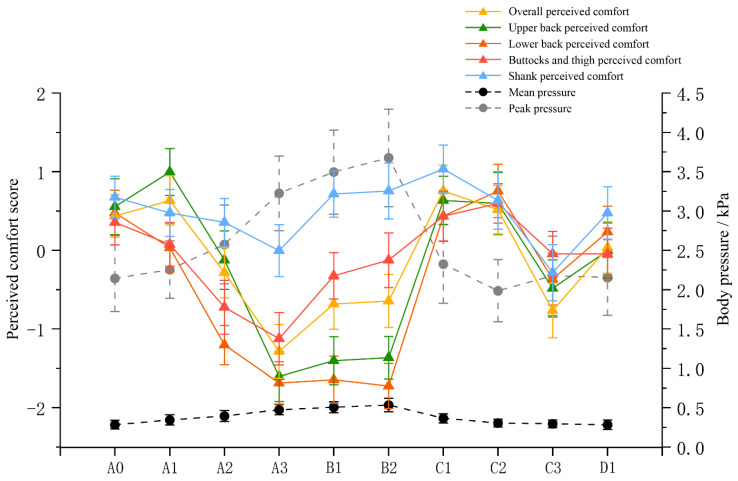
Variation in body pressure and perceived comfort in different typical conditions of bed boards.

We conducted tests on 400 participants in 10 experimental groups. We recorded the body pressure distribution and perceived comfort in typical bed board positions corresponding to each participant subgroup (Table 3). The results of the correlation analysis (see Table 4) showed that there were significant positive correlations between all types of comfort, with overall and back perceived comfort negatively correlated with mean and peak pressure; buttocks and thigh perceived comfort were only correlated with mean pressure, but shank comfort was not related to pressure indicators. We also used line graphs to present changes in body pressure and perceived comfort in different typical bed board conditions, and the results revealed that the trends of the perceived comfort and body pressure indicators were not always consistent (Figure 3). In terms of body pressure indicators, the trends of mean and peak pressures were basically the same. As the angle of raising the back bed board became larger, the mean and peak pressures increased (A0, A1, A2, A3), and all comfort indicators showed a decreasing trend, except for overall comfort and upper back comfort, which increased slightly at the beginning of the process. As the angle of raising the leg bed board increased (B1, B2), the rate of increase in the mean and peak pressures weakened, and the corresponding comfort indicators showed a certain positive correlation with the pressure indicators. As observed in the C1 and B2 conditions, with a 20° decrease in the back bed board, the pressure indicators decreased dramatically, and the comfort level was greatly improved. In the C2, C3, and D1 conditions, the amplitude of changes in mean and peak pressures decreased, and there was a roughly partial negative correlation between comfort performance and pressure indicators.

#### 3.2.2. Influence of Condition on Perceived Comfort

For both overall comfort and comfort at each site, the differences between the conditions were statistically significant. Table 3 displays the results of the analysis of variance (ANOVA) for the groups. Supplementary Table S1 presents the multiple comparison outcomes. In terms of perceived comfort, overall perceived comfort scores were highest in the C1 condition and lowest in the A3 condition. The localized comforts, in addition to showing some correlation with overall comfort, also revealed their own characteristics. For example, the shank comfort scores were higher in all conditions, and the comfort in the upper and lower back were lower in the A3, B1, and B2 conditions; upper back comfort peaked in the A1 condition, and lower back comfort peaked in the C2 condition. Consistent with overall comfort, all localized comfort scores reached their lowest values in the A3 condition.

#### 3.2.3. Multivariate Factors of Perceived Comfort

We used comfort as the dependent variable, and introduced individual factors for stepwise multiple linear regression to modulate the effects of pressure and posture on perceived comfort (Table 5), with the P-to-Enter significance level set at 0.05, and the P-to-Remove significance level set at 0.10, which implies that some of the variables will be excluded when the *p*-value is greater than 0.1. The relationship between pressure and comfort was significantly lower after adjusting for other variables, with only mean pressure having a statistically significant effect on buttocks and thigh perceived comfort (t = −2.207, *p* = 0.028). For the typical condition of the bed board, which is an unordered categorical variable, we set up dummy variables using the original typical condition as a reference, and all dummy variables were synchronized into the regression equation. The lying posture remained important in influencing comfort, and compared to the fully flat condition (A0), overall comfort and upper/lower back comfort were lower in the A2, A3, B1, B2, and C3 conditions, buttocks and thigh comfort were lower in the A2 and A3 conditions, and shank comfort was lower in the A3 and C3 conditions. In addition, height, weight, and BMI are common factors that impact overall comfort as well as the comfort of individual parts of the body. The greater the participant’s weight, the higher the comfort level was, whereas the higher the height and BMI, the lower the comfort level was. Among the participants in this experiment, the older the participant was, the lower the buttocks and thigh comfort scores were. The larger the contact area, the higher the comfort of the upper back, buttocks, and thigh.

## 4. Discussion

We examined the determinants of perceived comfort in smart bedding design, focusing on the effects of body pressure distribution and posture on perceived comfort in different smart bedding conditions, in addition to aspects such as age, height, weight, BMI, and other influencing factors. We expect our results to provide more comprehensive and accurate empirical evidence for the design of smart bedding to boost the public’s sleep health.

Our findings indicate that after adjusting for other factors, only mean pressure showed a negative correlation with buttocks and thigh comfort, which is consistent with the results of Zhang et al., who found that in the process of using smart beddings, the comfort of core body parts is related to mean pressure [39]. However, we also found that body pressure distribution and comfort did not always change in tandem, which is inconsistent with previous studies [25,30,40,41]. For example, Stanglmeier et al. [40] evaluated the biomechanical quality of sleeping postures based on the effect of the seat pan and backrest’s angles on the interface pressure distribution. Their findings showed that a decrease in the weighted average pressure level positively affected comfort and sleep quality. Ren et al. [25] indicated that the more favorable the pressure distribution of the mattress (i.e., the lower the peak pressure, average pressure, and pressure index), the higher the subjective comfort score. A possible reason for these results is that, in smart bedding design, changes in bed condition lead to instability in the users’ body postures, and they require additional muscle activity to maintain a stable body position, consequently resulting in stretching and fatigue of the back and buttocks muscles. Consequently, the body pressure distribution in the same condition does not always remain constant during smart bedding utilization [41]. Reflecting comfort through pressure monitoring at a single moment is not entirely accurate, particularly when comfort is influenced by other individual factors [39,42]. However, as stated by Ito and Usuki [27], equalizing the pressure as much as possible remains an effective way to improve comfort. Wu et al. established finite element models based on the mechanical properties of contact surfaces and body pressure distribution [43], which provide powerful tools for dynamically evaluating pressure distribution during the sleep process. In future studies, utilizing existing findings and tools to enhance the design of smart bedding to better monitor and timely adjust the pressure distribution will be an effective way to improve sleep comfort and sleep quality.

We found that sleep posture has consistently been a major influence on perceived comfort, even after adjusting for other factors, which aligns with the results of Park et al. [26]. Past studies have mentioned that a comfortable bed system that provides adequate musculoskeletal support is essential to achieve good quality sleep [44]. In this study, when one side of the smart bed head or foot was lifted too high without corresponding support on the other side, the comfort level dropped sharply, that is, the A3, B1, B2, and C3 conditions presented lower comfort. In the A3, B1, and B2 conditions, overall comfort, back comfort, and buttocks and thigh comfort were relatively low, whereas in the C3 condition, overall comfort and shank comfort were somewhat low. Consideration can be given to adding a linkage mechanism in future designs. This mechanism would adjust the leg board height proportionally when the back board is lifted or lowered, achieving relative body balance; this is consistent with previous findings [45]. In addition, back comfort was lower in the B1 and B2 conditions, and the body pressure distribution showed excessive pressure in the back area, whereas it improved significantly in the C1 condition. We speculate that setting the 60-degree limit value for the back bed board is not meaningful, and the range can be appropriately reduced to facilitate user operation. Furthermore, different sleep postures also have some effect on the contact surface temperature, which may be another intrinsic reason for comfort [46,47].

Moreover, the individual requirements of special groups—such as patients with pressure ulcers and obstructive sleep apnea—can affect sleep posture comfort. Pressure ulcers, medically termed “decubitus ulcers,” have always been a major threat to the rehabilitation of bedridden patients; the skin over bony prominences are considered to be pressure-vulnerable areas, and preventing the excessive accumulation of pressure in vulnerable areas is a common preventive and therapeutic approach [48,49]. Smart bedding can serve as a way to realize such functions based on pressure-sensing algorithms that determine the possibility of pressure ulcers by considering the pressure intensity and duration on specific body parts in different bed conditions [50], as well as by reducing pressure in vulnerable areas, thereby improving comfort. In the case of obstructive sleep apnea, the supine posture is a poor choice because of poor airway geometry and reduced lung volume [51]. These individual requirements should be considered in future studies, where the range and number of participants can be expanded to explore the implementation pathway of smart bedding in reducing health risks and improving health status.

Because anthropometric characteristics (such as height, weight, and BMI) are different for each individual, the comfort performance of smart bedding varies. We found that heavier people felt more comfortable using smart bedding. To some extent, greater body weight can make the bed conform more closely to one’s body contour, providing better support and pressure relief and reducing excessive pressure in certain areas. In addition, fat tissue can provide additional cushioning effects [52], keeping the smart bedding soft without losing its supportive quality. In contrast, thin people, whose bones are more prominent, lack this cushioning performance. We observed a large gap between the lower back area and the bed board, resulting in a small contact area and high mean pressure. When designing for thin people, it is necessary to increase the softness of the contact surface to enhance comfort. Smart bedding also provides poor comfort for taller individuals. This may be because the current experimental smart bedding has conventional specifications, and after the smart bedding panels are rotated, the support for the back and legs of taller people is insufficient. When designing for such people, the number and size of bed board divisions must be determined. Moreover, it was inferred that smart bedding provides poorer comfort in the buttocks and thigh areas when used by older adults. This may be related to the attenuation of core strength with age [53], resulting in higher comfort requirements in the core areas of the body and requiring additional attention when designing for older adults. In addition, other studies have reported significant differences between males and females in sensitivity to pressure on contact surfaces, which may also influence sleep posture preference and comfort [54]. Other anthropometric differences (such as pelvic size and flexibility) have also been influential [55]. Thus, these factors should be further explored in future studies.

Key summary of this study:

- The results of the study indicated that, after adjusting for other factors, only mean pressure showed a negative correlation with buttocks and thigh comfort.
- The body pressure distribution and comfort did not always change in tandem, possibly due to the fact that large changes in bed condition lead to instability in users’ body postures, requiring additional muscle activity. This consequently results in stretching and fatigue of the back and buttock muscles.
- Sleep posture has consistently been a major influence on perceived comfort.
- When the head or foot of the smart bed was lifted too high without corresponding support on the other side, the comfort level dropped sharply. Consideration can be given to adding a linkage mechanism in the next design. This mechanism would adjust the leg board height proportionally when the back board is lifted or lowered, achieving relative body balance.
- The variation in body pressure and comfort across different conditions of the smart bed is significant, and can prevent excessive accumulation of pressure in specific areas. It is feasible to explore the implementation pathway of smart bedding in reducing health risks and improving health status (such as pressure ulcers and obstructive sleep apnea) through special designs.

## 5. Strengths and Limitations

We adopted a two-way approach in which subjective and objective approaches can be contrasted, and the determinants of comfort can be effectively investigated and applied to smart bedding design. To the best of our knowledge, this is the first study to explore perceived comfort factors for smart bedding products. Owing to the diversity of smart adjustment methods, the boundary between seats and bedding has becoming increasingly blurred. Therefore, this study appropriately applies the more mature seat comfort research methods to bedding comfort research. Unlike existing studies on seating and bedding comfort, smart bedding products offer more application scenarios due to their unique posture adjustment and automated control methods. For instance, significant variations in body pressure and comfort across different postures of the smart bed suggest that empirical evidence-based design can prevent excessive pressure accumulation in specific areas. This approach is a viable pathway to reducing health risk and improving health status, an aspect not yet addressed in existing studies. These findings are expected to serve as an important basis for the bed board division, angle optimization, drive mode tuning, and automation control program setting in subsequent smart bed development. Additionally, they are expected to serve as a reference for the development of the smart bedding industry. However, our sample size was small, and convenience sampling resulted in some limitations in the characteristics of the included population, which may restrict the generalization of our findings. We will conduct long-term comfort studies with a broader variation in subjects’ age, geography, education, and other factors. Moreover, due to time and financial constraints, the variables considered were not comprehensive. Future studies should include other variables in psychological, social, demographic, and other dimensions to provide more comprehensive evidence. Additionally, the existing literature treats comfort and discomfort as two separate variables [56]. Therefore, utilizing different scales (one for comfort and one for discomfort) may yield more precise and meaningful results, and should be considered in future research.

## 6. Conclusions

In this study, we found that the comfort of smart bedding was partially correlated with body pressure distribution. Sleeping posture still has a major influence on comfort, making it necessary to optimize the range of bed board angles based on comfort performance to adjust sleeping posture. In addition, we should consider the special needs of different populations, such as those with issues that relate to height, weight, age, and health conditions. Therefore, the bed board division, mattress softness, and additional linkage mechanisms should be optimized based on traditional smart bedding to improve comfort and its effectiveness in reducing health risks and improving health status.

## Figures and Tables

**Table 1:** Typical conditions of the smart bed.

Typical Conditions	Angles (1 & 2, 2 & 3)	Schemas	Typical Conditions	Angles (1 & 2, 2 & 3)	Schemas
A0	0°, 0°	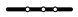	B2	60°, 40°	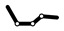
A1	20°, 0°	** 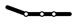 **	C1	40°, 40°	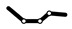
A2	40°, 0°	** 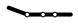 **	C2	20°, 40°	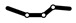
A3	60°, 0°	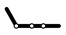	C3	0°, 40°	** 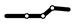 **
B1	60°, 20°	** 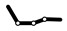 **	D1	0°, 20°	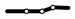

**Table 2:** Demographic traits of the participants.

Variable	Category	Mean or Frequency	Standard Deviation or Percentage (%)
Sex	Male	256	64.0
	Female	144	36.0
Age (years)		28.28	3.41
Weight (kg)		65.10	16.50
Height (cm)		169.36	9.13
BMI (kg/m^2^)		22.39	3.95

Note. BMI: Body Mass Index.

**Table 3:** Body pressure and perceived comfort in different typical conditions of bed boards.

	A0	A1	A2	A3	B1	B2	C1	C2	C3	D1	F *	*p* Value
Overall perceived comfort	0.44 (1.08)	0.64 (1.25)	−0.28 (1.28)	−1.28 (1.37)	−0.68 (1.28)	−0.64 (1.35)	0.76 (1.30)	0.52 (1.23)	−0.76 (1.39)	0.04 (1.31)	7.489	<0.000
Upper back perceived comfort	0.56 (1.42)	1.00 (1.19)	−0.12 (1.48)	−1.60 (1.26)	−1.40 (1.22)	−1.36 (1.08)	0.64 (1.22)	0.60 (1.58)	−0.48 (1.45)	0.00 (1.41)	12.745	<0.000
Lower back perceived comfort	0.48 (1.16)	0.04 (1.24)	−1.20 (1.00)	−1.68 (1.07)	−1.64 (1.19)	−1.72 (1.14)	0.44 (1.29)	0.76 (1.36)	−0.36 (1.32)	0.24 (1.30)	16.924	<0.000
Buttocks and thigh perceived comfort	0.36 (1.15)	0.08 (1.12)	−0.72 (1.37)	−1.12 (1.33)	−0.32 (1.18)	−0.12 (1.39)	0.44 (1.26)	0.60 (1.00)	−0.04 (1.14)	−0.04 (1.31)	4.595	<0.000
Shank perceived comfort	0.68 (1.07)	0.48 (1.19)	0.36 (1.22)	0.00 (1.32)	0.72 (1.17)	0.76 (1.42)	1.04 (1.21)	0.64 (1.47)	−0.28 (1.43)	0.48 (1.33)	2.234	0.021
Mean pressure (kPa)	0.29 (0.11)	0.35 (0.13)	0.40 (0.14)	0.48 (0.13)	0.51 (0.14)	0.54 (0.17)	0.37 (0.12)	0.31 (0.10)	0.30 (0.10)	0.29 (0.12)	13.848	<0.000
Peak pressure (kPa)	2.15 (0.85)	2.26 (0.73)	2.58 (1.00)	3.23 (0.95)	3.50 (1.07)	3.68 (1.24)	2.33 (1.00)	1.99 (0.79)	2.18 (1.01)	2.16 (0.96)	10.329	<0.000

* Analysis of variance (ANOVA).

**Table 4:** Correlation analysis between body pressure and perceived comfort.

	Overall Perceived Comfort	Upper Back Perceived Comfort	Lower Back Perceived Comfort	Buttocks and Thigh Perceived Comfort	Shank Perceived Comfort	Mean Pressure	Peak Pressure
Overall perceived comfort	1						
Upper back perceived comfort	0.759 **	1					
Lower back perceived comfort	0.645 **	0.689 **	1				
Buttocks and thigh perceived comfort	0.559 **	0.383 **	0.456 **	1			
Shank perceived comfort	0.467 **	0.319 **	0.218 **	0.549 **	1		
Mean pressure	−0.149 *	−0.237 **	−0.401 **	−0.133 *	0.077	1	
Peak pressure	−0.133 *	−0.219 **	−0.356 **	−0.075	0.095	0.726 **	1

* *p* < 0.05, the two were related; ** *p* < 0.01, the two were significantly correlated.

**Table 5:** Multiple linear regression (overall perceived comfort).

		Ratio	Standard Error	Standardized Regression Coefficient	t	*p* Value	95% Confidence Interval	
Overall perceived comfort ^1^	Height	−0.161	0.075	−1.029	−2.141	0.033	−0.309	−0.013
	Weight	0.191	0.099	2.200	1.933	0.054	−0.004	0.385
	BMI	−0.637	0.283	−1.763	−2.253	0.025	−1.195	−0.080
	Condition							
	A0				1.000			
	A1	0.190	0.367	0.040	0.518	0.605	−0.533	0.913
	A2	−0.831	0.405	−0.175	−2.052	0.041	−1.629	−0.033
	A3	−1.905	0.467	−0.400	−4.076	0.000	−2.826	−0.984
	B1	−1.264	0.462	−0.266	−2.737	0.007	−2.173	−0.354
	B2	−1.180	0.462	−0.248	−2.552	0.011	−2.091	−0.269
	C1	0.328	0.369	0.069	0.888	0.375	−0.399	1.055
	C2	0.108	0.362	0.023	0.300	0.765	−0.604	0.821
	C3	−1.192	0.360	−0.251	−3.308	0.001	−1.902	−0.482
	D1	0.360	−0.083	−1.097	0.274	−1.105	0.314	−0.395
Upper back perceived comfort ^2^	Height	−0.200	0.076	−1.146	−2.629	0.009	−0.351	−0.050
	Weight	0.250	0.100	2.580	2.501	0.013	0.053	0.447
	BMI	−0.852	0.287	−2.109	−2.973	0.003	−1.417	−0.288
	Contact area	0.000	0.000	0.370	2.705	0.007	0.000	0.001
	Condition							
	A0			1.000				
	A1	0.316	0.372	0.059	0.849	0.397	−0.417	1.048
	A2	−1.110	0.410	−0.209	−2.705	0.007	−1.918	−0.302
	A3	−2.845	0.473	−0.535	−6.009	0.000	−3.778	−1.912
	B1	−2.572	0.468	−0.484	−5.500	0.000	−3.494	−1.651
	B2	−2.474	0.468	−0.465	−5.280	0.000	−3.397	−1.551
	C1	−0.034	0.374	−0.006	−0.092	0.927	−0.771	0.702
	C2	0.050	0.366	0.009	0.136	0.892	−0.672	0.772
	C3	−1.048	0.365	−0.197	−2.870	0.004	−1.767	−0.328
	D1	−0.554	0.365	−0.104	−1.520	0.130	−1.273	0.164
Lower back perceived comfort ^3^	Sex	−0.649	0.240	−0.205	−2.708	0.007	−1.121	−0.177
	Height	−0.197	0.070	−1.181	−2.800	0.006	−0.335	−0.058
	Weight	0.215	0.092	2.328	2.331	0.021	0.033	0.396
	BMI	−0.617	0.264	−1.606	−2.338	0.020	−1.138	−0.097
	Condition							
	A0				1.000			
	A1	−0.323	0.343	−0.064	−0.942	0.347	−0.997	0.352
	A2	−1.417	0.378	−0.280	−3.748	0.000	−2.162	−0.672
	A3	−1.690	0.436	−0.334	−3.873	0.000	−2.549	−0.830
	B1	−1.593	0.431	−0.315	−3.697	0.000	−2.442	−0.744
	B2	−1.612	0.432	−0.318	−3.734	0.000	−2.462	−0.761
	C1	0.116	0.345	0.023	0.335	0.738	−0.563	0.794
	C2	0.301	0.338	0.059	0.892	0.373	−0.364	0.966
	C3	−0.812	0.336	−0.161	−2.415	0.017	−1.475	−0.150
	D1	−0.234	0.336	−0.046	−0.696	0.487	−0.896	0.428
Buttocks and thigh perceived comfort ^4^	Age	−0.062	0.027	−0.163	−2.309	0.022	−0.115	−0.009
	Height	−0.273	0.069	−1.906	−3.932	0.000	−0.410	−0.136
	Weight	0.343	0.091	4.332	3.776	0.000	0.164	0.522
	BMI	−1.113	0.261	−3.366	−4.267	0.000	−1.627	−0.599
	Contact area	0.004	0.001	0.602	2.768	0.006	0.001	0.006
	Mean pressure	−2.024	0.917	−0.239	−2.207	0.028	−3.830	−0.217
	Condition							
	A0				1.000			
	A1	−0.219	0.338	−0.050	−0.648	0.518	−0.886	0.447
	A2	−1.056	0.373	−0.243	−2.828	0.005	−1.792	−0.320
	A3	−1.458	0.431	−0.335	−3.385	0.001	−2.307	−0.609
	B1	−0.605	0.426	−0.139	−1.422	0.156	−1.444	0.233
	B2	−0.339	0.426	−0.078	−0.794	0.428	−1.179	0.501
	C1	0.177	0.340	0.041	0.521	0.603	−0.493	0.848
	C2	0.301	0.334	0.069	0.903	0.368	−0.356	0.958
	C3	−0.375	0.332	−0.086	−1.128	0.260	−1.030	0.280
	D1	0.332	−0.090	−1.180	0.239	−1.046	0.262	−0.392
Shank perceived comfort ^5^	Age	−0.114	0.028	−0.298	−4.083	0.000	−0.169	−0.059
	Height	−0.293	0.072	−2.031	−4.067	0.000	−0.435	−0.151
	Weight	0.400	0.094	5.010	4.239	0.000	0.214	0.586
	BMI	−1.267	0.271	−3.804	−4.681	0.000	−1.801	−0.734
	Condition							
	A0				1.000			
	A1	−0.280	0.351	−0.064	−0.798	0.426	−0.972	0.412
	A2	−0.585	0.388	−0.133	−1.509	0.133	−1.349	0.179
	A3	−1.113	0.447	−0.254	−2.488	0.014	−1.994	−0.232
	B1	−0.362	0.442	−0.083	−0.819	0.414	−1.232	0.509
	B2	−0.298	0.442	−0.068	−0.673	0.501	−1.170	0.574
	C1	0.281	0.353	0.064	0.795	0.427	−0.415	0.977
	C2	−0.034	0.346	−0.008	−0.099	0.921	−0.716	0.648
	C3	−0.967	0.345	−0.221	−2.805	0.005	−1.646	−0.288
	D1	−0.198	0.345	−0.045	−0.574	0.567	−0.877	0.481

F_1_ = 4.834, *p* < 0.000; adjusted R^2^ = 0.207; F_2_ = 8.858, *p* < 0.000; adjusted R^2^ = 0.349; F_3_ = 10.359, *p* < 0.000; adjusted R^2^ = 0.390; F_4_ = 4.544, *p* < 0.000; adjusted R^2^ = 0.195; F_5_ = 3.497, *p* < 0.000; adjusted R^2^ = 0.146, BMI = body mass index.

## Data Availability

The original contributions presented in the study are included in the article/supplementary material, and further inquiries can be directed to the corresponding author/s.

## Supplementary Materials

The following supporting information can be downloaded at: https://www.mdpi.com/article/10.3390/s24134058/s1, Table S1: Multiple comparison of perceived comfort in different typical conditions.
