# Supplementary material for: Determinants of Perceived Comfort: Multi-Dimensional Thinking in Smart Bedding Design
Source: Sensors (Basel). 2024 Jun 21;24(13):4058. doi: 10.3390/s24134058 (PMC11243896; doi:10.3390/s24134058)
Supplement: Supplementary file 1 [file sensors-24-04058-s001.zip › sensors-3020023-supplementary.pdf]

Table S1: Multiple comparison of perceived comfort in different typical conditions.

(I) Condition	(J) Condition	Mean Difference (I-J)	Std. Error	Sig.	95% Confidence Interval	
					Lower Bound	Upper Bound
Overall perceived comfort						
A0	A1	-.200	.358	1.000	-1.42	1.02
	A2	.720	.358	1.000	-.50	1.94
	A3	1.720	.358	.000	.50	2.94
	B1	1.120	.358	.127	-.10	2.34
	B2	1.080	.358	.182	-.14	2.30
	C1	-.320	.358	1.000	-1.54	.90
	C2	-.080	.358	1.000	-1.30	1.14
	C3	1.200	.358	.060	-.02	2.42
	D1	.400	.358	1.000	-.82	1.62
A1	A2	.920	.358	.701	-.30	2.14
	A3	1.920	.358	.000	.70	3.14
	B1	1.320	.358	.018	.10	2.54
	B2	1.280	.358	.027	.06	2.50
	C1	-.120	.358	1.000	-1.34	1.10
	C2	.120	.358	1.000	-1.10	1.34
	C3	1.400	.358	.007	.18	2.62
	D1	.600	.358	1.000	-.62	1.82
A2	A3	1.000	.358	.365	-.22	2.22
	B1	.400	.358	1.000	-.82	1.62
	B2	.360	.358	1.000	-.86	1.58
	C1	-1.040	.358	.259	-2.26	.18
	C2	-.800	.358	1.000	-2.02	.42
	C3	.480	.358	1.000	-.74	1.70
	D1	-.320	.358	1.000	-1.54	.90
A3	B1	-.600	.358	1.000	-1.82	.62
	B2	-.640	.358	1.000	-1.86	.58
	C1	-2.040	.358	.000	-3.26	-.82
	C2	-1.800	.358	.000	-3.02	-.58
	C3	-.520	.358	1.000	-1.74	.70
	D1	-1.320	.358	.018	-2.54	-.10
B1	B2	-.040	.358	1.000	-1.26	1.18
	C1	-1.440	.358	.005	-2.66	-.22
	C2	-1.200	.358	.060	-2.42	.02

	C3	.080	.358	1.000	-1.14	1.30
	D1	-.720	.358	1.000	-1.94	.50
B2	C1	-1.400	.358	.007	-2.62	-.18
	C2	-1.160	.358	.087	-2.38	.06
	C3	.120	.358	1.000	-1.10	1.34
	D1	-.680	.358	1.000	-1.90	.54
C1	C2	.240	.358	1.000	-.98	1.46
	C3	1.520	.358	.002	.30	2.74
	D1	.720	.358	1.000	-.50	1.94
C2	C3	1.280	.358	.027	.06	2.50
	D1	.480	.358	1.000	-.74	1.70
C3	D1	-.800	.358	1.000	-2.02	.42
Upper back perceived comfort						
A0	A1	-.440	.375	1.000	-1.72	.84
	A2	.680	.375	1.000	-.60	1.96
	A3	2.160	.375	.000	.88	3.44
	B1	1.960	.375	.000	.68	3.24
	B2	1.920	.375	.000	.64	3.20
	C1	-.080	.375	1.000	-1.36	1.20
	C2	-.040	.375	1.000	-1.32	1.24
	C3	1.040	.375	.393	-.24	2.32
	D1	.560	.375	1.000	-.72	1.84
A1	A2	1.120	.375	.204	-.16	2.40
	A3	2.600	.375	.000	1.32	3.88
	B1	2.400	.375	.000	1.12	3.68
	B2	2.360	.375	.000	1.08	3.64
	C1	.360	.375	1.000	-.92	1.64
	C2	.400	.375	1.000	-.88	1.68
	C3	1.480	.375	.007	.20	2.76
	D1	1.000	.375	.538	-.28	2.28
A2	A3	1.480	.375	.007	.20	2.76
	B1	1.280	.375	.049	.00	2.56
	B2	1.240	.375	.071	-.04	2.52
	C1	-.760	.375	1.000	-2.04	.52
	C2	-.720	.375	1.000	-2.00	.56
	C3	.360	.375	1.000	-.92	1.64
	D1	-.120	.375	1.000	-1.40	1.16

A3	B1	-.200	.375	1.000	-1.48	1.08
	B2	-.240	.375	1.000	-1.52	1.04
	C1	-2.240	.375	.000	-3.52	-.96
	C2	-2.200	.375	.000	-3.48	-.92
	C3	-1.120	.375	.204	-2.40	.16
	D1	-1.600	.375	.002	-2.88	-.32
B1	B2	-.040	.375	1.000	-1.32	1.24
	C1	-2.040	.375	.000	-3.32	-.76
	C2	-2.000	.375	.000	-3.28	-.72
	C3	-.920	.375	.979	-2.20	.36
	D1	-1.400	.375	.015	-2.68	-.12
B2	C1	-2.000	.375	.000	-3.28	-.72
	C2	-1.960	.375	.000	-3.24	-.68
	C3	-.880	.375	1.000	-2.16	.40
	D1	-1.360	.375	.023	-2.64	-.08
C1	C2	.040	.375	1.000	-1.24	1.32
	C3	1.120	.375	.204	-.16	2.40
	D1	.640	.375	1.000	-.64	1.92
C2	C3	1.080	.375	.285	-.20	2.36
	D1	.600	.375	1.000	-.68	1.88
C3	D1	-.480	.375	1.000	-1.76	.80
Lower back perceived comfort						
A0	A1	.440	.338	1.000	-.71	1.59
	A2	1.680	.338	.000	.53	2.83
	A3	2.160	.338	.000	1.01	3.31
	B1	2.120	.338	.000	.97	3.27
	B2	2.200	.338	.000	1.05	3.35
	C1	.040	.338	1.000	-1.11	1.19
	C2	-.280	.338	1.000	-1.43	.87
	C3	.840	.338	.893	-.31	1.99
	D1	.240	.338	1.000	-.91	1.39
A1	A2	1.240	.338	.019	.09	2.39
	A3	1.720	.338	.000	.57	2.87
	B1	1.680	.338	.000	.53	2.83
	B2	1.760	.338	.000	.61	2.91
	C1	-.400	.338	1.000	-1.55	.75
	C2	-.720	.338	1.000	-1.87	.43

	C3	.400	.338	1.000	-.75	1.55
	D1	-.200	.338	1.000	-1.35	.95
A2	A3	.480	.338	1.000	-.67	1.63
	B1	.440	.338	1.000	-.71	1.59
	B2	.520	.338	1.000	-.63	1.67
	C1	-1.640	.338	.000	-2.79	-.49
	C2	-1.960	.338	.000	-3.11	-.81
	C3	-.840	.338	.893	-1.99	.31
	D1	-1.440	.338	.002	-2.59	-.29
A3	B1	-.040	.338	1.000	-1.19	1.11
	B2	.040	.338	1.000	-1.11	1.19
	C1	-2.120	.338	.000	-3.27	-.97
	C2	-2.440	.338	.000	-3.59	-1.29
	C3	-1.320	.338	.008	-2.47	-.17
	D1	-1.920	.338	.000	-3.07	-.77
B1	B2	.080	.338	1.000	-1.07	1.23
	C1	-2.080	.338	.000	-3.23	-.93
	C2	-2.400	.338	.000	-3.55	-1.25
	C3	-1.280	.338	.012	-2.43	-.13
	D1	-1.880	.338	.000	-3.03	-.73
B2	C1	-2.160	.338	.000	-3.31	-1.01
	C2	-2.480	.338	.000	-3.63	-1.33
	C3	-1.360	.338	.005	-2.51	-.21
	D1	-1.960	.338	.000	-3.11	-.81
C1	C2	-.320	.338	1.000	-1.47	.83
	C3	.800	.338	1.000	-.35	1.95
	D1	.200	.338	1.000	-.95	1.35
C2	C3	1.120	.338	.069	-.03	2.27
	D1	.520	.338	1.000	-.63	1.67
C3	D1	-.600	.338	1.000	-1.75	.55
Buttocks and thigh perceived comfort						
A0	A1	.280	.353	1.000	-.92	1.48
	A2	1.080	.353	.159	-.12	2.28
	A3	1.480	.353	.002	.28	2.68
	B1	.680	.353	1.000	-.52	1.88
	B2	.480	.353	1.000	-.72	1.68
	C1	-.080	.353	1.000	-1.28	1.12

	C2	-.240	.353	1.000	-1.44	.96
	C3	.400	.353	1.000	-.80	1.60
	D1	.400	.353	1.000	-.80	1.60
A1	A2	.800	.353	1.000	-.40	2.00
	A3	1.200	.353	.050	.00	2.40
	B1	.400	.353	1.000	-.80	1.60
	B2	.200	.353	1.000	-1.00	1.40
	C1	-.360	.353	1.000	-1.56	.84
	C2	-.520	.353	1.000	-1.72	.68
	C3	.120	.353	1.000	-1.08	1.32
	D1	.120	.353	1.000	-1.08	1.32
A2	A3	.400	.353	1.000	-.80	1.60
	B1	-.400	.353	1.000	-1.60	.80
	B2	-.600	.353	1.000	-1.80	.60
	C1	-1.160	.353	.075	-2.36	.04
	C2	-1.320	.353	.015	-2.52	-.12
	C3	-.680	.353	1.000	-1.88	.52
	D1	-.680	.353	1.000	-1.88	.52
A3	B1	-.800	.353	1.000	-2.00	.40
	B2	-1.000	.353	.324	-2.20	.20
	C1	-1.560	.353	.001	-2.76	-.36
	C2	-1.720	.353	.000	-2.92	-.52
	C3	-1.080	.353	.159	-2.28	.12
	D1	-1.080	.353	.159	-2.28	.12
B1	B2	-.200	.353	1.000	-1.40	1.00
	C1	-.760	.353	1.000	-1.96	.44
	C2	-.920	.353	.632	-2.12	.28
	C3	-.280	.353	1.000	-1.48	.92
	D1	-.280	.353	1.000	-1.48	.92
B2	C1	-.560	.353	1.000	-1.76	.64
	C2	-.720	.353	1.000	-1.92	.48
	C3	-.080	.353	1.000	-1.28	1.12
	D1	-.080	.353	1.000	-1.28	1.12
C1	C2	-.160	.353	1.000	-1.36	1.04
	C3	.480	.353	1.000	-.72	1.68
	D1	.480	.353	1.000	-.72	1.68
C2	C3	.640	.353	1.000	-.56	1.84

	D1	.640	.353	1.000	-.56	1.84
C3	D1	.000	.353	1.000	-1.20	1.20
Shank perceived comfort						
A0	A1	.200	.373	1.000	-1.07	1.47
	A2	.320	.373	1.000	-.95	1.59
	A3	.680	.373	1.000	-.59	1.95
	B1	-.040	.373	1.000	-1.31	1.23
	B2	-.080	.373	1.000	-1.35	1.19
	C1	-.360	.373	1.000	-1.63	.91
	C2	.040	.373	1.000	-1.23	1.31
	C3	.960	.373	.703	-.31	2.23
	D1	.200	.373	1.000	-1.07	1.47
A1	A2	.120	.373	1.000	-1.15	1.39
	A3	.480	.373	1.000	-.79	1.75
	B1	-.240	.373	1.000	-1.51	1.03
	B2	-.280	.373	1.000	-1.55	.99
	C1	-.560	.373	1.000	-1.83	.71
	C2	-.160	.373	1.000	-1.43	1.11
	C3	.760	.373	1.000	-.51	2.03
	D1	.000	.373	1.000	-1.27	1.27
A2	A3	.360	.373	1.000	-.91	1.63
	B1	-.360	.373	1.000	-1.63	.91
	B2	-.400	.373	1.000	-1.67	.87
	C1	-.680	.373	1.000	-1.95	.59
	C2	-.280	.373	1.000	-1.55	.99
	C3	.640	.373	1.000	-.63	1.91
	D1	-.120	.373	1.000	-1.39	1.15
A3	B1	-.720	.373	1.000	-1.99	.55
	B2	-.760	.373	1.000	-2.03	.51
	C1	-1.040	.373	.377	-2.31	.23
	C2	-.640	.373	1.000	-1.91	.63
	C3	.280	.373	1.000	-.99	1.55
	D1	-.480	.373	1.000	-1.75	.79
B1	B2	-.040	.373	1.000	-1.31	1.23
	C1	-.320	.373	1.000	-1.59	.95
	C2	.080	.373	1.000	-1.19	1.35
	C3	1.000	.373	.517	-.27	2.27

	D1	.240	.373	1.000	-1.03	1.51
B2	C1	-.280	.373	1.000	-1.55	.99
	C2	.120	.373	1.000	-1.15	1.39
	C3	1.040	.373	.377	-.23	2.31
	D1	.280	.373	1.000	-.99	1.55
C1	C2	.400	.373	1.000	-.87	1.67
	C3	1.320	.373	.031	.05	2.59
	D1	.560	.373	1.000	-.71	1.83
C2	C3	.920	.373	.947	-.35	2.19
	D1	.160	.373	1.000	-1.11	1.43
C3	D1	-.760	.373	1.000	-2.03	.51
